# Determining Optimal Intervals for In-Person Visits During Video-Based Telemedicine Among Patients With Hypertension: Cluster Randomized Controlled Trial

**DOI:** 10.2196/45230

**Published:** 2023-06-08

**Authors:** Yuji Nishizaki, Haruo Kuroki, So Ishii, Shigeyuki Ohtsu, Chizuru Watanabe, Hiroto Nishizawa, Masashi Nagao, Masanori Nojima, Ryo Watanabe, Daisuke Sato, Kensuke Sato, Yumi Kawata, Hiroo Wada, Goichiro Toyoda, Katsumi Ohbayashi

**Affiliations:** 1 Division of Medical Education Juntendo University School of Medicine Tokyo Japan; 2 Medical Technology Innovation Center Juntendo University Tokyo Japan; 3 Sotobo Children's Clinic Chiba Japan; 4 Kudan-Shita Eki-Mae CoCo Clinic Tokyo Japan; 5 Nakanoshima Diabetes Clinic Kanagawa Japan; 6 Akasaka Odayaka Clinic Tokyo Japan; 7 Minamisunamachi Odayaka Clinic Tokyo Japan; 8 Odayaka Life Medical Clinic Saitama Japan; 9 Medical Corporation Junreikai Tokyo Japan; 10 Center for Translational Research, The Institute of Medical Science The University of Tokyo Tokyo Japan; 11 Graduate School of Health Innovation Kanagawa University of Human Services Kanagawa Japan; 12 Center for Next Generation of Community Health Chiba University Hospital Chiba Japan; 13 Clinical Research and Trial Center Juntendo University Hospital Tokyo Japan; 14 Department of Public Health Juntendo University Graduate School of Medicine Tokyo Japan; 15 Medley, Inc Tokyo Japan; 16 Ohbayashi Clinic Tochigi Japan

**Keywords:** hypertension, Japan, lost productivity time, patient satisfaction, telemedicine

## Abstract

**Background:**

Introducing telemedicine in outpatient treatment may improve patient satisfaction and convenience. However, the optimal in-person visit interval for video-based telemedicine among patients with hypertension remains unreported in Japan.

**Objective:**

We determined the optimal in-person visit interval for video-based telemedicine among patients with hypertension.

**Methods:**

This was a cluster randomized controlled noninferiority trial. The target sites were 8 clinics in Japan that had a telemedicine system, and the target patients were individuals with essential hypertension. Among patients receiving video-based telemedicine, those who underwent in-person visits at 6-month intervals were included in the intervention group, and those who underwent in-person visits at 3-month intervals were included in the control group. The follow-up period of the participants was 6 months. The primary end point of the study was the change in systolic blood pressure, and the secondary end points were the rate of treatment continuation after 6 months, patient satisfaction, health economic evaluation, and safety evaluation.

**Results:**

Overall, 64 patients were enrolled. Their mean age was 54.5 (SD 10.3) years, and 60.9% (39/64) of patients were male. For the primary end point, the odds ratio for the estimated difference in the change in systolic blood pressure between the 2 groups was 1.18 (90% CI –3.68 to 6.04). Notably, the criteria for noninferiority were met. Patient satisfaction was higher in the intervention group than in the control group. Furthermore, the indirect costs indicated that lost productivity was significantly lesser in the intervention group than in the control group. Moreover, the treatment continuation rate did not differ between the intervention and control groups, and there were no adverse events in either group.

**Conclusions:**

Blood pressure control status and safety did not differ between the intervention and control groups. In-person visits at 6-month intervals may cause a societal cost reduction and improve patient satisfaction during video-based telemedicine.

**Trial Registration:**

UMIN Clinical Trials Registry (UMIN-CTR) UMIN000040953; https://tinyurl.com/2p8devm9

## Introduction

The introduction of telemedicine in outpatient treatment may improve patient satisfaction and convenience [1,2]. Moreover, it can overcome several challenges related to in-person visits in outpatient care. One of the challenges in treating lifestyle-related diseases in outpatient clinics is treatment dropout [3]. A widespread use of telemedicine can help prevent such treatment dropouts among working people. Notably, the COVID-19 pandemic has caused many interruptions in medical care. For example, in a survey of 30,000 Japanese workers, the treatment of 11% of the patients requiring regular hospital visits was interrupted during the pandemic [4]. Under these circumstances, the wide use of telemedicine is expected to prevent the interruption of medical visits owing to the pandemic.

Many studies in the relevant literature have reported the effectiveness of telemedicine [5-9]. In Japan, a previous study involving a 1-year follow-up of patients with hypertension randomized into 2 groups (standard care or telemedicine) revealed that the mean weekly systolic blood pressure at the end of the study was significantly lower in the telemedicine group [10]. However, telemedicine has not been widely adopted in Japan, partly because of the national medical fee system. Moreover, in Japan, reimbursement for telemedicine is <50% of the reimbursement for normal in-person visits [11]. Considering that the standard reimbursement for telemedicine is equal to or greater than the reimbursement for in-person visits worldwide, lesser reimbursement in the Japanese fee system is a major challenge. Another challenge is the interval between in-person visits. Until March 2022, in-person visits were required once every 3 months for insured patients who were using telemedicine in Japan [11]. This short interval between in-person visits may be a barrier to the widespread use of telemedicine in Japan. Since April 2022, the restrictions on in-person visit intervals in telemedicine have been relaxed, and the obligation of conducting such visits every 3 months has been abolished; in addition, there are no longer any restrictions on in-person visit intervals. However, this change was not based on evidence-based medicine but was largely attributed to the COVID-19 pandemic.

To our knowledge, there are no studies in Japan on the optimal in-person visit interval for telemedicine in patients with hypertension based on a multifaceted evaluation, including health economics and patient satisfaction assessments. Therefore, this study’s aim was to generate evidence regarding the optimal in-person visit intervals for patients with essential hypertension during the video-based telemedicine.

## Methods

### Study Design

This was a cluster randomized controlled trial conducted as a noninferiority trial.

### Site Selection

A total of 8 clinics in Japan with a video-based telemedicine system at study initiation were included in this study. Notably, a clinic was defined as a place where physicians practiced medicine for the public, or a specified number of people who did not have facilities for admitting patients or had facilities for admitting ≤19 patients.

### Study Population

Study participants were included in each clinic. The inclusion criteria were as follows: adult patients receiving outpatient telemedicine or who were about to start receiving outpatient telemedicine; those diagnosed with essential hypertension and prescribed with antihypertensive medication for ≥3 months; those with stable hypertension (ie, no change in the antihypertensive medication prescription for over 3 months) and stable comorbidities; those who could visit outpatient departments in the third and sixth months after enrollment; and those who provided their free written consent to participate in the study after receiving a full explanation of the study requirements. The exclusion criteria were as follows: patients with drug allergies; patients who were pregnant; patients with visual impairment or other problems that could interfere with telemedicine; patients with end-stage renal failure; patients with cancer who were receiving anticancer drug therapy; patients with chronic respiratory diseases, such as obstructive lung disease, who were receiving home oxygen therapy; patients participating in other clinical trials; patients who required frequent visits to the hospital for blood tests to manage comorbidities; and patients whose participation was deemed medically or scientifically inappropriate by the principal investigator and coinvestigators.

During patient recruitment, the study purpose and details were explained using an informed consent form. Patients who provided informed consent were enrolled. Moreover, we ensured that patients could withdraw their consent even after participating in the research.

### Procedures

Among patients receiving video-based telemedicine, those who underwent in-person visits at 6-month intervals were included in the intervention group, and those who underwent in-person visits at 3-month intervals were included in the control group. Stratified cluster randomization was performed using clinic location (23 wards of Tokyo [urban] vs outside the 23 wards of Tokyo [suburban]) and the target number of cases as allocation factors.

Prescriptions for antihypertensive medications could be changed during the study period as needed at the discretion of the physician in charge. Moreover, medication status was assessed through self-report at the time of enrollment and at follow-up in both groups.

### Patient Enrollment and Follow-up Period

Physicians recruited the patients during their first in-person visits. After recruitment by a physician, each patient spoke with a clinical research assistant who used an explanatory document to outline the study to the patient. The originally planned patient enrollment period was 3 months, but the COVID-19 pandemic led to some delays; hence, the patient enrollment period was extended to 8 months. The registration period for the first half group (3 clinics) was from May 29, 2020, to January 31, 2021, and that for the second half group (5 clinics) was from July 31, 2020, to March 31, 2021. Notably, the follow-up period for both groups was 6 months. Follow-up was planned for the 3rd and 6th months after patient enrollment.

### Blood Pressure Measurement

Blood pressure was measured using an upper-arm digital automated sphygmomanometer (HEM-8712) from Omron in both intervention and control groups. Two blood pressure measurements were taken at rest in a sitting position for each patient. The interval between the first and second measurements was ≥2 minutes, and the average of the 2 measured values was used for this study. During the remote examination, the patient reported the blood pressure measurements taken on the day of the examination through video, and the doctor confirmed the blood pressure values visually.

### End Points

The primary end point of the study was the change in systolic blood pressure (6-month value – baseline value). Secondary end points were the treatment continuation rate at 6 months, patient satisfaction ratings (Ministry of Health, Labor, and Welfare [MHLW] survey for behavior at the outpatient visit [Question 15; Multimedia Appendix 1], and EuroQol 5 Dimensions 5 Level [EQ-5D-5L]), a health economic evaluation, and a safety evaluation (adverse events). Furthermore, we examined whether the patient attended in-person visits for hypertension management outside the scheduled timing.

### Health Economics Evaluation Questionnaire

Although the health care costs are expected to be lower for telemedicine than for normal in-person visits, their clinical outcomes may be comparable. Therefore, we performed a cost-minimization analysis of telemedicine (intervention group) versus normal in-person visits (control group). The analysis was conducted from the perspective of public health care based on the Guidelines for Analysis of Cost-Effectiveness Evaluation in the Central Social Insurance Medical Council, 2nd edition [12]. Given that the use of telemedicine may directly affect patient productivity, an additional analysis was performed to include productivity loss in terms of cost using a patient questionnaire (Multimedia Appendices 2 and 3), which comprised questions regarding employment status, occupation, and annual income.

### Patients’ Backgrounds

At patient enrollment, in addition to blood pressure, the following data were collected: sex, age, height, weight, pulse rate, the presence of dyslipidemia, the presence of diabetes, smoking and alcohol consumption status, family history (hypertension in parents), and use of antihypertensive medications. Patient satisfaction and health economic evaluation questionnaires were administered at enrollment and at 3- and 6-month follow-up examinations.

### Sample Size Calculation

The target sample size of the study was 70. If the SD of blood pressure after the antihypertensive medication is considered 7.9 mm Hg based on the report by Chow et al [13], the SD of the change in systolic blood pressure is 7.9 mm Hg when the correlation coefficient between the pre- and postvalues is set at 0.5. Based on this assumption, when the noninferiority margin was set at –5.0 mm Hg, the required sample size was determined to be 64 cases (1-sided α=.05, power 80%). The choice of –5.0 mm Hg as the margin for our work was based on a discussion by 3 internal medicine specialists. The sample size of the *t* test was used for the above calculations because the assumption of 0 for the intracluster correlation is equivalent to that for the *t* test. The dropout rate was set at 10% (3 patients each in the intervention and control groups), and the target total number of patients was set at 70. If the correlation coefficient between the pre- and postvalues was >0.5, the SD reduced, thereby reducing the required sample size.

### Statistical Analysis

Using the baseline and 6-month data, we used the time point, allocation group, and their interaction terms as the explanatory variables in a linear mixed model, whereas the systolic blood pressure (ie, the mean of two measurements) was used as the response variable. In this model, the change from the baseline value (ie, the primary end point) was estimated as the effect of the interaction term. Further, the covariates sex, age, and baseline blood pressure values were estimated as fixed effects, and the interaction term between the baseline blood pressure values and time points, allocation factors, and cluster were estimated as random effects. Kenward–Roger method was used to estimate the degrees of freedom. Further, variance components were specified for the variance–covariance structure of the random effects. First, the correlation structure of time points among participants was specified as unstructured, but because the model did not converge, it was specified as first-order autoregressive (1) [14]. A *P* value of <.05 was considered statistically significant. All statistical analyses apart from the health economic evaluation were performed using SAS (version 9.4; SAS Institute).

### Data Management

An electronic data capturing system, REDCap (Research Electronic Data Capture), was used for data management [15].

### Ethical Approval

This study was approved by the Ethics Committee of Juntendo University Hospital (reception 20-038) and was conducted in accordance with the principles of the Declaration of Helsinki. The study was registered in the University Hospital Medical Information Network (UMIN) Clinical Trials Registry system [UMIN Clinical Trials Registry (UMIN-CTR)]. The UMIN study ID was UMIN000040953.

This study enrolled patients who fully understood the study purpose, signed a written consent form, and were willing to participate. Patient anonymity is maintained in this paper, including in the text, tables, and figures.

## Results

### Overview

No clinic participating in this clinical trial had the capacity to admit more than 20 patients. Patients in the intervention group visited 5 clinics, including 2 and 3 in urban and suburban areas, respectively. Patients in the control group visited the other 3 clinics, including 1 and 2 in urban and suburban areas, respectively. Overall, 31 and 33 patients were included in the intervention and control groups, respectively. However, 1 patient in the control group dropped out of the study before the 3-month visit after withdrawing consent. The mean age of participants was 54.5 (SD 10.3) years, and 60.9% (39/64) of the participants were male. Patient characteristics, including diabetes, dyslipidemia, smoking, alcohol drinking, and family history of hypertension, did not significantly differ between the 2 groups. However, the use of antihypertensive medication was significantly different between these groups. Table 1 summarizes the patient characteristics. Trends in blood pressure in both groups are shown in Table 2.

**Table 1:** Patient characteristics.

Characteristics			All patients (n=64)		Telemedicine practice group (intervention group) (n=31)		In-person visit group (control group) (n=33)		*P* value
Age (years), mean (SD)			54.5 (10.3)		51.5 (7.4)		57.2 (11.9)		.02^a^
Male, n (%)			39 (60.9)		17 (54.8)		22 (66.7)		.44
Height (cm), mean (SD)			165.4 (9.2)		165.2 (9.4)		165.7 (9.1)		.79
Weight (kg), mean (SD)			71.0 (17.0)		71.6 (18.6)		70.5 (15.6)		.74
Pulse rate per minute (bpm), mean (SD)			73.3 (11.9)		73.8 (11.2)		72.8 (12.7)		.74
Diabetes, n (%)			4 (6.3)		2 (6.5)		2 (6.1)		>.99
Dyslipidemia, n (%)			25 (39.1)		12 (38.7)		13 (39.4)		>.99
Smoking (current smoker), n (%)			10 (15.6)		3 (9.7)		7 (21.2)		.31
Alcohol drinking, n (%)			41 (64.1)		21 (67.7)		20 (60.6)		.77
Family history of hypertension, n (%)			26 (40.6)		16 (51.6)		10 (30.3)		.19
Drug adherence (complied with physicians’ instructions), n (%)			61 (95.3)		29 (93.5)		32 (97)		.60
**Antihypertensive medication, n (%)**									
	ACE-I^b^	2 (3.1)		0 (0)		2 (6.1)		.49	
	ARB^c^	25 (39.1)		15 (48.4)		10 (30.3)		.20	
	CCB^d^	35 (54.7)		8 (25.8)		27 (81.8)		<.001^a^	
	β-blocker	4 (6.3)		2 (6.5)		2 (6.1)		>.99	
	α-blocker	0 (0)		0 (0)		0 (0)		>.99	
	Diuretics	5 (7.8)		5 (16.1)		0 (0)		.02^a^	
	Combination drug	16 (25)		13 (41.9)		3 (9.1)		.003^a^	
	ARB+CCB	15 (23.4)		12 (38.7)		3 (9.1)		.007^a^	
	ARB+diuretics	1 (1.6)		1 (3.2)		0 (0)		.48	

^a^*P*<.05.

^b^ACE-I: angiotensin-converting enzyme inhibitor.

^c^ARB: angiotensin II receptor blocker.

^d^CCB: calcium-channel blocker.

**Table 2:** Trends in blood pressure.

		All patients (n=64), mean (SD)	Telemedicine practice group (Intervention group) (n=31), mean (SD)	In-person visit group (Control group) (n=33), mean (SD)	*P* value
**At time of registration (mm Hg)**					
	Systolic blood pressure	132.6 (14.3)	128.8 (13.1)	136.3 (14.7)	.03^a^
	Diastolic blood pressure	87.3 (9.3)	85.3 (9.6)	89.2 (8.8)	.09
**Follow-up at 3 months** **(mm Hg)^b^**					
	Systolic blood pressure	129.8 (13.7)	125.4 (10.8)	134.1 (15.0)	.01^a^
	Diastolic blood pressure	86.0 (8.9)	82.2 (6.6)	89.7 (9.4)	<.001^a^
**Follow-up at 6 months** **(mm Hg)^b^**					
	Systolic blood pressure	133.7 (12.1)	130.9 (10.8)	136.4 (12.9)	.07
	Diastolic blood pressure	87.4 (9.2)	85.6 (10.2)	89.2 (7.9)	.12

^a^*P*<.05.

^b^The number of patients at 3- and 6-month follow-ups was 63 because 1 patient in the control group withdrew from this study.

### Primary End Points

**Figure 1:**
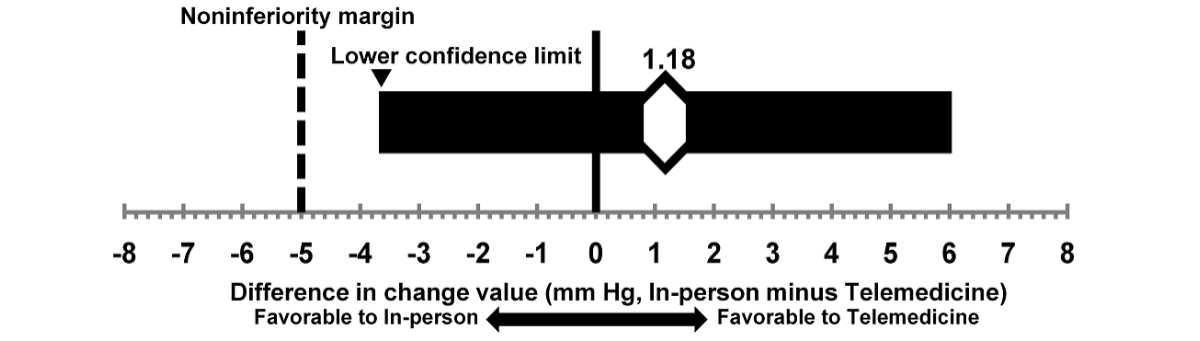
Differences in the change in systlic blood pressure estimated using linear mixed model. Lower confidence limit (one-sided 95%) of the difference exceeded noninferiority margin of -5 mm Hg. The point estimate was odds ratio (OR) 1.18 (90% CI -3.68 to 6.04).

**Figure 2:**
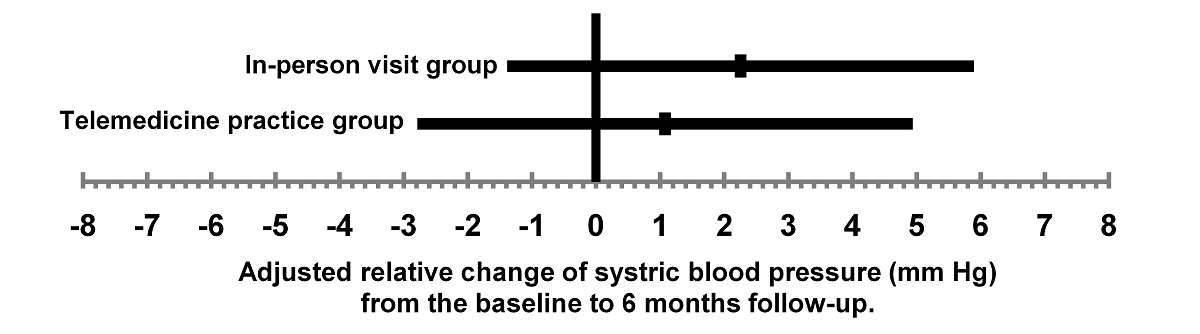
The adjusted relative change in systolic blood pressure estimated using a linear mixed model. The short vertical lines centered in the long lines represent the point estimates. Long lines indicate the 90% CI of the point estimates.

A linear mixed model was used to estimate the difference in the change in systolic blood pressure between the 2 groups (odds ratio [OR] 1.18, 90% CI –3.68 to 6.04). The lower limit of the CI was the noninferiority margin of –5, which met the criteria for noninferiority in this study (Figure 1). Additionally, 1 patient in the intervention group made a nonscheduled in-person visit for hypertension management. Nonetheless, even after this patient was excluded from the analysis, the criterion for noninferiority was met, with a between-group (intervention and control group) difference (OR 0.51, 90% CI –4.68 to 5.69). Figure 2 shows the adjusted relative change in systolic blood pressure estimated by the linear mixed model.

### Secondary End Points

There were no differences in the treatment continuation rate at the 6-month follow-up between the intervention (31/31, 100%) and control (32/33, 97%) groups (*P*=.51). Moreover, there were no adverse events in either group. There was no significant difference in terms of in-person visits for hypertension management at unscheduled times between the 2 groups (only 1 patient in the intervention and no patients in the control group; *P*=.48). The results of patient satisfaction are shown in Table 3. Although there was no difference in the EQ-5D-5L results between the two groups, patient satisfaction was partially higher in the intervention group than in the control group, as assessed by the MHLW survey for behavior at the outpatient visit (question 15). In particular, responses to questions about consultation time and conversation with the physician showed that patient satisfaction was higher in the intervention group. A boxplot graph of the MHLW survey results is shown in Figure S1 in Multimedia Appendix 4. The raw data regarding patient satisfaction are shown in Table S1 in Multimedia Appendix 5. The health economic evaluation results indicated no significant difference in the 3-month average direct medical costs between the 2 groups. The indirect costs (calculated by converting lost productivity hours into Japanese Yen) indicated significantly lower lost productivity in the intervention group than in the control group (Table 4).

The number of patients at the 3- and 6-month follow-ups was 63 because 1 patient in the control group withdrew from this study.

**Table 3:** Difference in the change in patient satisfaction (n=63).

		Estimated difference in change value^a^ (control group minus intervention group)	95% CI (lower limit to upper limit)	*P* value
**EuroQol 5 Dimensions 5 Level**				
	Anxiety and depression	0.08	–0.14 to 0.30	.51
	Mobility	0.02	–0.09 to 0.12	.72
	Pain/discomfort	–0.06	–0.21 to 0.10	.54
	Self-care	N/A^b^	N/A	N/A
	Usual activities	–0.03	–0.09 to 0.03	.37
	Visual analog scale	–4.46	–9.32 to 0.40	.12
**Ministry of Health, Labor, and Welfare survey for behavior at the outpatient visit (question 15)**				
	Q1. Are you satisfied with the waiting time for consultation?	0.18	–0.11 to 0.47	.30
	Q2. Are you satisfied with the consultation time?	0.29	0.09 to 0.48	.01^c^
	Q3. Are you satisfied with the content of the medical examination and treatment provided by the physician?	0.14	–0.04 to 0.32	.19
	Q4. Are you satisfied with conversation with the physician?	0.21	0.04 to 0.37	.04^c^
	Q5. Are you satisfied with the hospital staff other than physicians?	0.23	0.03 to 0.43	.05
	Q6. Are you satisfied with the privacy protection measures during the consultation?	0.19	–0.25 to 0.64	.37
	Q7. Overall, are you satisfied with this hospital?	0.30	0.14 to 0.46	.002^c^

^a^These results were estimated by linear mixed model.

^b^N/A: not available.

^c^*P*<.05.

**Table 4:** Health economics evaluation (¥1=US $0.0074).

		All patients (n=64)	Telemedicine practice group (intervention group) (n=31)	In-person visit group (control group) (n=33)	*P* value
**Direct medical cost (**¥**), mean (SD)**					
	At the time of registration	7561.7 (4461.3)	7674.8 (3404.7)	7455.45 (5319.1)	.84
	Follow-up at 3 months	5006.8 (2048.5)	4568.4 (2357.9)	5431.56 (1623.0)	.09
	Follow-up at 6 months	8189.1 (4513.8)	9131.0 (4589.7)	7276.56 (4314.6)	.10
**Indirect costs calculated by converting lost productivity hours into Yen (**¥**), mean (SD)**					
	At the time of registration	32,734.8 (33,372.6)	31,733.1 (33,202.2)	33,675.78 (34,018.8)	.81
	Follow-up at 3 months	18,728.6 (24,435.6)	9102.3 (13,082.6)	27,771.52 (28,999.6)	.002^a^
	Follow-up at 6 months	28,170.9 (28,174.7)	26,918.0 (26,157.0)	29,347.80 (30,305.9)	.73
**Societal costs (total of direct medical costs and indirect costs) (**¥**), mean (SD)**					
	At the time of registration	40,391.4 (33,574.4)	39,441.9 (33,343.5)	41,283.30 (34,282.0)	.82
	Follow-up at 3 months	23,697.5 (25,030.7)	13,670.7 (14,319.0)	33,116.62 (29,195.6)	.001^a^
	Follow-up at 6 months	36,292.6 (28,756.4)	36,065.1 (26,242.5)	36,506.35 (31,341.6)	.95

^a^*P*<.05.

## Discussion

### Overview

The results of this study indicated no difference in blood pressure between the intervention and control groups. The health economics evaluation results showed that costs were lower for the intervention group than for the control group when productivity losses (social costs) were considered. Furthermore, patient satisfaction, mainly with consultation time and conversation with the physician, was higher in the telemedicine group.

Blood pressure values differed between the 2 groups at registration, and this could have affected the primary end point, although the outcome was the change in systolic blood pressure from baseline. The systolic blood pressure at registration was adjusted in a statistical model while evaluating the primary end point as per major guidelines, such as the European Medicines Agency guidelines [16]. Regarding patient background, the medications used were different among all patients (Table 1). Moreover, physician preferences for antihypertensive drug prescriptions may have varied among the participating clinics. This bias may be attributed to cluster randomization with an insufficient number of clusters. However, the primary end point of the study was the stability of systolic blood pressure in real world, regardless of the medication used, and this study demonstrated noninferiority of the primary end point in patients with stable essential hypertension. Therefore, the 3-month follow-up did not necessarily have to be an in-person visit, suggesting that, under certain conditions, continued treatment is also possible with in-person visits at 6-month intervals during video-based telemedicine.

Doctor-patient communication is crucial for improving both health outcomes and treatment adherence in patients. However, physicians tend to interrupt patients’ complaints in an outpatient setting [17]. Moreover, insufficient doctor-patient communication was recognized by patients as a critical cause of treatment dissatisfaction [18]. In this study, in the patient satisfaction evaluation through the MHLW survey for behavior at outpatient visits (question 15), the intervention group reported greater satisfaction with consultation time and conversation with the physician. The satisfaction with consultation time may be attributed to the better accessibility of web-based treatment. Notably, good accessibility is a convenient and major factor in increasing patient satisfaction, and this has been a particularly important factor during the COVID-19 pandemic [19,20]. The intervention group reported higher scores in satisfaction with the conversation with the doctor, which may indicate characteristics of telemedicine as it is a one-to-one interaction with a doctor (in a private room) that allows for a more in-depth and pleasant conversation with the doctor than is possible in a regular outpatient clinic. Moreover, increasing patient satisfaction can have a positive effect on health outcomes [21]. The results of the question “Are you satisfied with the waiting time for consultation?” were not significantly different between the two groups. The reasons behind the answers to this question may differ according to the operational system of each medical facility. Therefore, the specific implications of these results are not clear from this question. Further studies are required to address these issues.

The relationship between telemedicine and cost-effectiveness has been reported in various clinical fields [22-25]. For instance, the report of the Victorian Stroke Telemedicine intervention contained an estimation of the cost per quality-adjusted life year (QALY) gained from stroke telemedicine. At 12 months, the QALYs were estimated to be 0.43 per person in the control period and 0.5 per person in the intervention period. After 1000 bootstrapping iterations, the Victorian Stroke Telemedicine intervention period, compared with the control period, was more effective and cost saving in 50.6% of iterations and cost-effective (US $0 and US $33,357.9 per QALY gained) in 10.4% of iterations, potentially contributing to the further implementation of telemedicine for acute stroke care in Australia [25]. In this study, a comparison of societal costs, which are the sum of productivity losses and direct medical costs converted into monetary values, revealed that the costs were lower in the intervention group than in the control group. Notably, the calculation of productivity losses included patient and family transportation expenses and labor productivity loss (workforce productivity loss). These results indicate that telemedicine is effective in reducing societal costs after accounting for productivity losses. To our knowledge, this is the first study to report the usefulness of video-based telemedicine for societal cost reduction. In addition to noninferiority in blood pressure control, safety, and higher patient satisfaction of telemedicine versus in-person visits, the reduction in societal costs suggests that telemedicine is more cost-effective in a society-based analysis. Thus, telemedicine is valuable from health economics and medical perspective.

This study has some limitations. First, the number of participating facilities was small; hence, the data obtained from 8 included facilities may not represent overall telemedicine in Japan. Second, the study cohort was limited to patients with stable essential hypertension. Therefore, the study results may not be generalizable. Third, the COVID-19 pandemic posed difficulty in patient recruitment; hence, the patient recruitment period was extended from 3 to 8 months. Consequently, the seasons for patient registration varied, and the difference in season may have affected blood pressure [26]. Fourth, there was 1 unscheduled patient visit for hypertension management in the intervention group, which possibly influenced the blood pressure findings by affecting the change in systolic blood pressure. However, the noninferiority criteria in this study were met even after excluding this patient. Fifth, the observation period was short. We have only demonstrated the usefulness of video-based telemedicine for at least 6 months of follow-up. It would be desirable to evaluate the usefulness of the video-based telemedicine in clinical trials with follow-up periods of 6 months or more. Sixth, the study did not directly assess the prevention of cardiovascular events. The original and primary purpose of blood pressure control is to prevent cardiovascular events [27]. A study design with cardiovascular events as the primary end point is desirable but not feasible because a long observation period is required to evaluate cardiovascular events. Further studies are required to address this issue. Seventh, the timing of the start of outpatient telemedicine could affect the patient satisfaction rating. In this study, we did not evaluate the relationship between the patient satisfaction and timing of the start of the outpatient telemedicine. In future work, we would like to consider the mean time from the start of the outpatient telemedicine.

### Conclusions

Switching in-person visit intervals from 3 to 6 months in patients with stable essential hypertension did not cause any difference in the status of blood pressure control or safety in video-based telemedicine. Moreover, the use of video-based telemedicine is expected to have a social societal cost reduction effect and improve patient satisfaction.

## Appendix

Multimedia Appendix 1 Ministry of Health, Labor, and Welfare (MHLW) survey for behavior at the outpatient visit (Question 15).

Multimedia Appendix 2 Questionnaire for patients to investigate the health care economic impact of telemedicine (at the time of registration).

Multimedia Appendix 3 Questionnaire for patient to investigate the health care economic impact of telemedicine (3-month and 6-month follow-ups).

Multimedia Appendix 4 The results of the Ministry of Health, Labor, and Welfare (MHLW) survey for patients satisfaction.

Multimedia Appendix 5 Raw data for patient satisfaction.

Multimedia Appendix 6 CONSORT-eHEALTH checklist (V 1.6.1).

## Abbreviations

EQ-5D-5L: EuroQol 5 Dimensions 5 Level
MHLW: Ministry of Health, Labor, and Welfare
OR: odds ratio
QALY: quality-adjusted life year
REDCap: Research Electronic Data Capture
UMIN: University Hospital Medical Information Network

## Data Availability

Because we did not obtain consent from patients for their data to be shared publicly, the data are not available. The corresponding author will respond to inquiries on the data analysis in this study.
