# Supplementary material for: Determining Optimal Intervals for In-Person Visits During Video-Based Telemedicine Among Patients With Hypertension: Cluster Randomized Controlled Trial
Source: JMIR Cardio. 2023 Jun 8;7:e45230. doi: 10.2196/45230 (PMC10288346; doi:10.2196/45230)
Supplement: Multimedia Appendix 1 [file cardio_v7i1e45230_app1.docx]

**Appendix 1.** Ministry of Health, Labor, and Welfare (MHLW) survey for behavior at the outpatient visit (Question 15)

Question 1. Are you satisfied with the waiting time for consultation?

Item 1. Very satisfied

Item 2. Slightly satisfied

Item 3. Average

Item 4. Slightly dissatisfied

Item 5. Very dissatisfied

Item 6. Others

Question 2. Are you satisfied with the consultation time?

Item 1. Very satisfied

Item 2. Slightly satisfied

Item 3. Average

Item 4. Slightly dissatisfied

Item 5. Very dissatisfied

Item 6. Others

Question 3. Are you satisfied with the content of the medical examination and treatment provided by the physician?

Item 1. Very satisfied

Item 2. Slightly satisfied

Item 3. Average

Item 4. Slightly dissatisfied

Item 5. Very dissatisfied

Item 6. Others

Question 4. Are you satisfied with conversation with the physician?

Item 1. Very satisfied

Item 2. Slightly satisfied

Item 3. Average

Item 4. Slightly dissatisfied

Item 5. Very dissatisfied

Item 6. Others

Question 5. Are you satisfied with the hospital staff other than physicians?

Item 1. Very satisfied

Item 2. Slightly satisfied

Item 3. Average

Item 4. Slightly dissatisfied

Item 5. Very dissatisfied

Item 6. Others

Question 6. Are you satisfied with the privacy protection measures during the consultation?

Item 1. Very satisfied

Item 2. Slightly satisfied

Item 3. Average

Item 4. Slightly dissatisfied

Item 5. Very dissatisfied

Item 6. Others

Question 7. Overall, are you satisfied with this hospital?

Item 1. Very satisfied

Item 2. Slightly satisfied

Item 3. Average

Item 4. Slightly dissatisfied

Item 5. Very dissatisfied

Item 6. Others
