# Supplementary material for: Determining Optimal Intervals for In-Person Visits During Video-Based Telemedicine Among Patients With Hypertension: Cluster Randomized Controlled Trial
Source: JMIR Cardio. 2023 Jun 8;7:e45230. doi: 10.2196/45230 (PMC10288346; doi:10.2196/45230)
Supplement: Multimedia Appendix 2 [file cardio_v7i1e45230_app2.docx]

**Appendix 2.** Questionnaire for patients to investigate the health care economic impact of telemedicine (at the time of registration)

1) Patient burdens

1. Travel expenses for hospital visits (round trip) (yen)
2. Travel time for hospital visits (round trip) (minute)
3. Consultation hours (minute)
4. Total time associated with medical examination (minute)

2) About the patient himself/herself

1. Are you currently working? (Yes/No)
2. If you are working, please answer the following questions:

a) What type of work do you perform? (Full-time/Other)

b) What is the industrial classification of your company? (Choose the one that best fits your needs):

Agriculture; Forestry; Fishing; Mining; Construction; Manufacturing; Electricity, gas, heat supply, and water supply; Information and communication; Transportation; Wholesale and retail; Finance and insurance; Real estate; Restaurant and lodging; Medical and welfare; Education and learning support; Complex service business; Service industry (not elsewhere classified)

3) About the family member accompanying the patient

1. Do you have a family member accompanying you? (Yes/No)

If you answered “yes,” please answer the following questions:

1. What is the sex of your family member(s) accompanying you? (Male/Female)
2. How old is the family member accompanying you?
3. Is the family member accompanying you currently working? (Yes/No)
4. If the family member accompanying you is working, please answer the following questions:

a) What type of work does he/she perform? (Full-time/Other)

b) What is the industrial classification of your company? (Choose the one that best fits your needs):

Agriculture; Forestry; Fishing; Mining; Construction; Manufacturing; Electricity, gas, heat supply, and water supply; Information and communication; Transportation; Wholesale and retail; Finance and insurance; Real estate; Restaurant and lodging; Medical and welfare; Education and learning support; Complex service business; Service industry (not elsewhere classified)

4) Circumstances of your visit

1. Time required for family members to accompany and care for the patient (minute)
2. Expenses for nursing care services (actual expenses) (yen)

For example, day care services, home nursing, etc.
