# Supplementary material for: Determining Optimal Intervals for In-Person Visits During Video-Based Telemedicine Among Patients With Hypertension: Cluster Randomized Controlled Trial
Source: JMIR Cardio. 2023 Jun 8;7:e45230. doi: 10.2196/45230 (PMC10288346; doi:10.2196/45230)
Supplement: Multimedia Appendix 3 [file cardio_v7i1e45230_app3.docx]

**Appendix 3.** Questionnaire for patient to investigate the health care economic impact of telemedicine (3-month and 6-month follow-ups).

1) Patient burdens

1. Travel expenses for hospital visits (round trip) (yen)
2. Travel time for hospital visits (round trip) (minute)
3. Consultation hours (minute)
4. Total time associated with medical examination (minute)
