# Supplementary figures and images for: Determining Optimal Intervals for In-Person Visits During Video-Based Telemedicine Among Patients With Hypertension: Cluster Randomized Controlled Trial
Source: JMIR Cardio. 2023 Jun 8;7:e45230. doi: 10.2196/45230 (PMC10288346; doi:10.2196/45230)

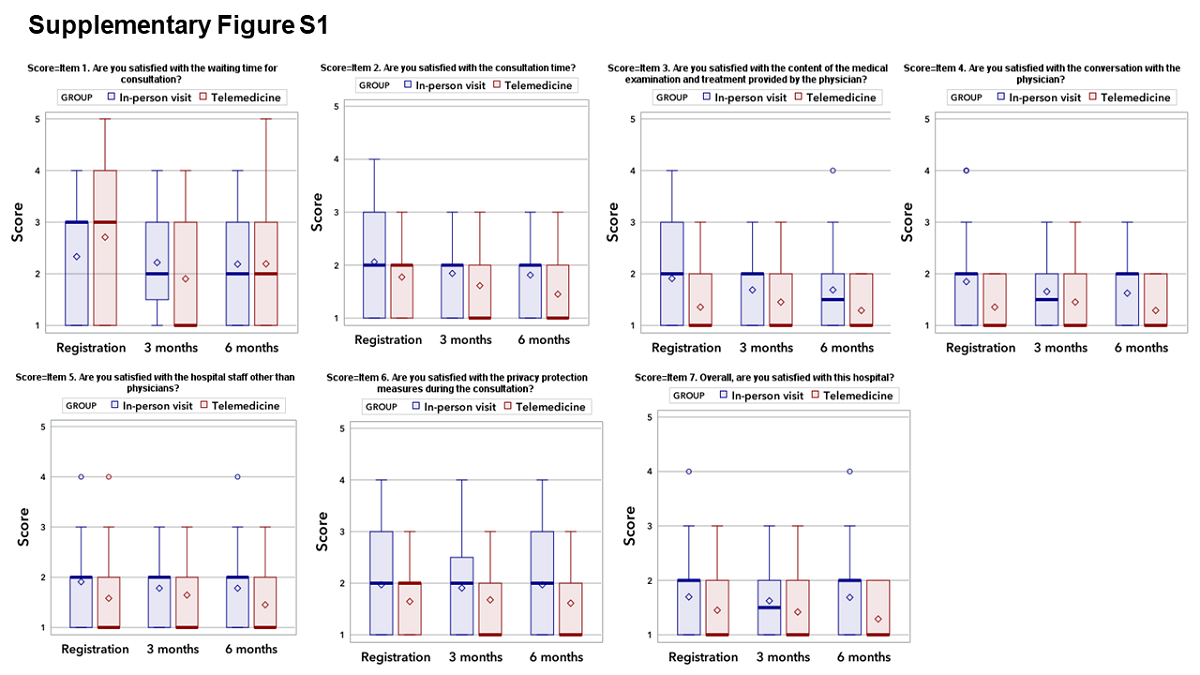

Supplement: Multimedia Appendix 4 [file cardio_v7i1e45230_app4.png]
