# Supplementary material for: Determining Optimal Intervals for In-Person Visits During Video-Based Telemedicine Among Patients With Hypertension: Cluster Randomized Controlled Trial
Source: JMIR Cardio. 2023 Jun 8;7:e45230. doi: 10.2196/45230 (PMC10288346; doi:10.2196/45230)
Supplement: Multimedia Appendix 5 [file cardio_v7i1e45230_app5.docx]

**Supplementary Table S1.** Raw data for patient satisfaction

|  |  | All (n = 64) | | Telemedicine  practice group  (Intervention group)  (n = 31) | | In-person visit group  (Control group)  (n = 33) | |  |
| --- | --- | --- | --- | --- | --- | --- | --- | --- |
|  | Timing of  evaluation | Average | SD | Average | SD | Average | SD | *P*-value |
| EuroQol 5 Dimensions 5 Level (EQ-5D-5L) |  |  |  |  |  |  |  |  |
| Anxiety/Depression (AD) | Registration | 1.29 | 0.61 | 1.33 | 0.71 | 1.25 | 0.51 | 0.59 |
|  | Follow-up  3 months | 1.17 | 0.38 | 1.19 | 0.40 | 1.16 | 0.37 | 0.70 |
|  | Follow-up  6 months | 1.16 | 0.41 | 1.13 | 0.34 | 1.19 | 0.48 | 0.54 |
| Mobility (MB) | Registration | 1.03 | 0.18 | 1.00 | 0.00 | 1.06 | 0.24 | 0.16 |
|  | Follow-up  3 months | 1.05 | 0.21 | 1.03 | 0.18 | 1.06 | 0.25 | 0.58 |
|  | Follow-up  6 months | 1.03 | 0.18 | 1.03 | 0.18 | 1.03 | 0.18 | 0.98 |
| Pain/Discomfort (PD) | Registration | 1.35 | 0.63 | 1.42 | 0.72 | 1.28 | 0.52 | 0.38 |
|  | Follow-up  3 months | 1.30 | 0.53 | 1.32 | 0.54 | 1.28 | 0.52 | 0.75 |
|  | Follow-up  6 months | 1.24 | 0.53 | 1.29 | 0.64 | 1.19 | 0.40 | 0.44 |
| Self-Care (SC) | Registration | 1.02 | 0.13 | 1.03 | 0.18 | 1.00 | 0.00 | 0.30 |
|  | Follow-up  3 months | 1.00 | 0.00 | 1.00 | 0.00 | 1.00 | 0.00 | NA |
|  | Follow-up  6 months | 1.00 | 0.00 | 1.00 | 0.00 | 1.00 | 0.00 | NA |
| Usual Activities (UA) | Registration | 1.09 | 0.29 | 1.10 | 0.30 | 1.09 | 0.29 | 0.93 |
|  | Follow-up  3 months | 1.08 | 0.27 | 1.03 | 0.18 | 1.13 | 0.34 | 0.17 |
|  | Follow-up  6 months | 1.05 | 0.21 | 1.06 | 0.25 | 1.03 | 0.18 | 0.54 |
| VAS (Visual Analog Scale) |  |  |  |  |  |  |  |  |
|  | Registration | 79.72 | 11.52 | 78.23 | 11.22 | 81.12 | 11.79 | 0.31 |
|  | Follow-up  3 months | 81.86 | 11.06 | 83.39 | 10.10 | 80.38 | 11.89 | 0.28 |
|  | Follow-up  6 months | 81.87 | 10.89 | 83.58 | 10.29 | 80.22 | 11.37 | 0.22 |
| Ministry of Health, Labor, and Welfare (MHLW) survey for behavior at the outpatient visit (Question 15) | | | | | | | | |
| Item 1. Are you satisfied with the waiting time for consultation? | Registration | 2.52 | 1.20 | 2.71 | 1.37 | 2.33 | 0.99 | 0.21 |
|  | Follow-up  3 months | 2.06 | 1.03 | 1.90 | 1.11 | 2.22 | 0.94 | 0.22 |
|  | Follow-up  6 months | 2.19 | 1.08 | 2.19 | 1.17 | 2.19 | 1.00 | 0.98 |
| Item 2. Are you satisfied with the consultation time? | Registration | 1.92 | 0.90 | 1.77 | 0.76 | 2.06 | 1.00 | 0.20 |
|  | Follow-up  3 months | 1.73 | 0.75 | 1.61 | 0.72 | 1.84 | 0.77 | 0.22 |
|  | Follow-up 6 months | 1.63 | 0.75 | 1.45 | 0.68 | 1.81 | 0.78 | 0.05 |
| Item 3. Are you satisfied with the content of the medical examination and treatment provided by the physician? | Registration | 1.64 | 0.82 | 1.35 | 0.55 | 1.91 | 0.95 | 0.006* |
|  | Follow-up  3 months | 1.57 | 0.71 | 1.45 | 0.68 | 1.69 | 0.74 | 0.19 |
|  | Follow-up  6 months | 1.49 | 0.69 | 1.29 | 0.46 | 1.69 | 0.82 | 0.02* |
| Item 4. Are you satisfied with the conversation with the physician? | Registration | 1.61 | 0.77 | 1.35 | 0.49 | 1.85 | 0.91 | 0.009* |
|  | Follow-up  3 months | 1.56 | 0.69 | 1.45 | 0.62 | 1.66 | 0.75 | 0.24 |
|  | Follow-up  6 months | 1.46 | 0.59 | 1.29 | 0.46 | 1.63 | 0.66 | 0.02* |
| Item 5. Are you satisfied with the hospital staff other than physicians? | Registration | 1.75 | 0.82 | 1.58 | 0.76 | 1.91 | 0.84 | 0.10 |
|  | Follow-up  3 months | 1.71 | 0.77 | 1.65 | 0.75 | 1.78 | 0.79 | 0.48 |
|  | Follow-up  6 months | 1.62 | 0.73 | 1.45 | 0.62 | 1.78 | 0.79 | 0.07 |
| Item 6. Are you satisfied with the privacy protection measures during the consultation? | Registration | 1.81 | 0.81 | 1.65 | 0.71 | 1.97 | 0.88 | 0.11 |
|  | Follow-up  3 months | 1.79 | 0.86 | 1.68 | 0.79 | 1.91 | 0.93 | 0.29 |
|  | Follow-up  6 months | 1.79 | 0.81 | 1.61 | 0.72 | 1.97 | 0.86 | 0.07 |
| Item 7. Overall, are you satisfied with this hospital? | Registration | 1.58 | 0.69 | 1.45 | 0.57 | 1.70 | 0.77 | 0.15 |
|  | Follow-up  3 months | 1.52 | 0.67 | 1.42 | 0.62 | 1.63 | 0.71 | 0.22 |
|  | Follow-up  6 months | 1.49 | 0.64 | 1.29 | 0.46 | 1.69 | 0.74 | 0.01* |
| NA = not available, SD = standard deviation, **P* < 0.05. | | | | | | | | |
